# Adjuvant chemotherapy in older women (ACTION) study – what did we learn from the pilot phase?

**DOI:** 10.1038/bjc.2011.377

**Published:** 2011-10-11

**Authors:** R Leonard, R Ballinger, D Cameron, P Ellis, L Fallowfield, M Gosney, L Johnson, L S Kilburn, A Makris, J Mansi, M Reed, A Ring, A Robinson, P Simmonds, G Thomas, J M Bliss

**Affiliations:** 1 Department of Surgery and Cancer, Imperial College London, London W12 0HS, UK; 2 Brighton and Sussex Medical School Falmer, Brighton BN1 9QG, UK; 3 University of Edinburgh Cancer Research Centre, Western General Hospital, Edinburgh EH4 2XU, UK; 4 Department of Medical Oncology, Guy's and St Thomas' Hospital and King's College London Biomedical Research Centre, London SE1 9RT, UK; 5 Clinical Health Sciences, University of Reading, White Knights, Reading RG6 6AH, UK; 6 ICR-Clinical Trials and Statistics Unit, The Institute of Cancer Research, 15 Cotswold Road, Sutton, Surrey SM2 5NG, UK; 7 Department of Clinical Oncology, Mount Vernon Hospital, Rickmansworth Road, Northwood, Middlesex HA6 2RN, UK; 8 Department of Surgery, University of Sheffield, Western Bank, Sheffield S10 2TN, UK; 9 Department of Clinical Oncology, Royal Sussex County Hospital, Eastern Road, Brighton BN2 5BE, UK; 10 Department of Clinical Oncology, Southend General Hospital, Prittlewell Chase Westcliff-on-Sea, Essex SS0 0RY; 11 Department of Clinical Oncology, Southampton General Hospital, Tremona Road, Southampton SO16 6YD, UK

**Keywords:** chemotherapy, elderly breast cancer, clinical trial

## Abstract

**Background:**

The ACTION trial was initiated to provide evidence from a randomised trial on the effects of chemotherapy in women aged over 70 years where evidence for risk and benefit are lacking.

**Methods:**

This was a randomised, phase III clinical trial for high risk, oestrogen receptor (ER) negative/ER weakly positive early breast cancer. The trial planned to recruit 1000 women aged 70 years and older, randomised to receive 4 cycles of anthracycline chemotherapy or observation. The primary endpoint was relapse-free interval. The trial included a pilot phase to assess the acceptability and feasibility of recruitment.

**Results:**

The trial opened at 43 UK centres. Information on number of patients approached was available from 38 centres. Of the 43 eligible patients that were approached, 39 were not randomised due to patients declining entry. After 10 months only 4 patients had been randomised and after discussion with the research funder, the trial was closed and funding terminated.

**Conclusion:**

Despite widespread support at several public meetings, input from patient groups including representation on the Trial Management Group, the trial failed to recruit due to the inability to convince patients to accept randomisation. It would therefore seem that randomising the patients to receive chemotherapy *vs* observation is not a viable design in the current era for this patient population.

Breast cancer is the most common malignancy in women in the western world, affecting one in nine women at some time in her life, with over 41 000 new cases and 13 000 deaths each year in the United Kingdom alone. In 2006, 33% (12 532) of new cases in the United Kingdom were in women aged 70 or over (Héry *et al*, 2008).

The increased incidence is primarily as a result of an ageing population and the changing lifestyle factors of that population, for example, later age at first birth, with the median age of onset being ∼65 years. Projected population forecasts in Western countries indicate that the proportion of older women will increase dramatically over the next 50 years (Yancik, 1997; Vercelli *et al*, 1998), which will result in a significant increase in the number of older women diagnosed with breast cancer. However, data from the EUROCARE II study (Vercelli *et al*, 1998) show that patients aged 65 to 99 diagnosed with breast cancer had a relative risk of cancer-related death at 1 year of 1.7 compared with those aged 55–64, although this reduced to 1.09 at 5 years. The reduction in mortality is therefore not uniform across all age ranges, with the 50% of women who present with the disease over the age of 70 years appearing to benefit least from recent advances (Bouchardy *et al*, 2007).

Endocrine therapy, with either tamoxifen or aromatase inhibitors, is routinely used for older patients with hormone responsive disease. Clinical trials are currently examining age-related variations in surgical care and radiotherapy (Turner *et al*, 1999; Wyld *et al*, 2004). However, the best management for older patients with high-risk disease, which is not hormone responsive, remains uncertain. The general observation of improvements in survival with respect to breast cancer are essentially confined to women aged <70 (Autier *et al*, 2011). It may not be coincidental that this age-associated cancer-specific survival is mirrored by the use of cytotoxic chemotherapy for early stage disease in younger patients.

With an average life expectancy of 15.3 years for a 70-year-old woman in the United Kingdom (Government Actuary's Department, 2006), the potential to gain a significant number of life years amply justifies research aimed at better targeting of chemotherapy treatment. Despite this, clinical trials evaluating adjuvant chemotherapy in this age group are sparse. A study of patients enrolled in 164 Southwest Oncology Group trials in the United States (Hutchins *et al*, 1999) found that patients aged 65 and over were underrepresented in trials, with only 9% of patients enrolled in breast cancer trials aged over 65, despite 49% of breast cancer patients being in that age group. The findings were similar when trials excluding older patients were omitted from the analysis.

## Adjuvant chemotherapy for early stage breast cancer

The EBCTCG overview of 60 trials of prolonged polychemotherapy *vs* no chemotherapy involving 36 000 women has confirmed that, adjuvant anthracycline-based chemotherapy reduces the annual odds of death by 38% (s.e.=5), for women under 50 years of age at diagnosis and by 20% (s.e.=4) in women aged 50–69 years of age (EBCTCG, 2005). A reduction in recurrence emerges chiefly during the first 5 years of follow-up, whereas the survival advantage grows throughout the first 10 years. Subgroup analyses of these data have provided further information about relative benefit of treatment by age and oestrogen receptor (ER) status.

Estimates for those aged over 70 years appear consistent with those for women aged 60–69 years, however, as only 1200 women are included in the published meta-analysis, firm conclusions cannot be drawn. Moreover, the observed association with age may be confounded by other differences, notably the increasing proportion of patients with ER-positive tumours.

However more recent research has identified a sub-population of elderly women with ER-positive disease who are at high risk of relapse (Durbecq *et al*, 2008), particularly those whose tumours show amplifications of HER2.

The lack of reliable trial data has led to a great disparity in the use of cytotoxic chemotherapy in early breast cancer patients aged over 70. There are no agreed protocols for selecting patients whether ER negative or ER positive with other risk factors, yet control of breast cancer is the most likely determinant of survival where co-morbid disease is limited (Clarke, 2006). Studies have shown that treatment is often less intensive in older patients irrespective of co-morbidity (Yancik *et al*, 1989; Bergmann *et al*, 1991; Martin *et al*, 1996; Lavelle *et al*, 2007), suggesting that perceived frailty rather than specific co-morbidities may have a role in determining the use of chemotherapy for which a lack of efficacy data could further influence the treatment decision.

The ACTION study was conceived out of an international interest in optimising treatment for older patients, and was to be one study within a comprehensive international portfolio run via the Breast International Group, EORTC, the International Breast Cancer Study Group and the International Collaborative Cancer Group, addressing the main question of whether older patients should be offered chemotherapy.

## Patients and methods

**Figure 1:**
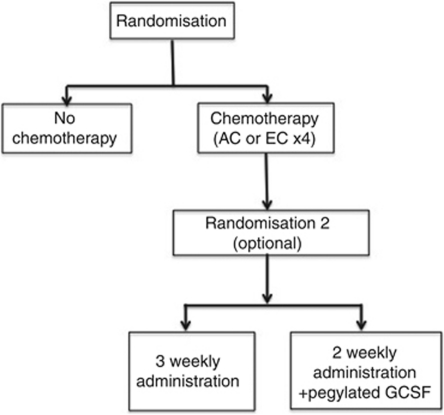
Trial design.

The ACTION Trial was a randomised controlled trial testing whether adjuvant chemotherapy (either doxorubicin 60 mg m^−2^ or epirubicin (90 mg m^−2^ plus cyclophosphamide 600 mg m^−2^ given thrice weekly for four cycles) improves the outcome in older women with high risk, ER negative/ER weakly positive breast cancer. An optional second randomisation comparing standard 3-weekly therapy with accelerated 2-weekly therapy with GCSF support aimed to test the acceptability and tolerability of this regimen within this patient group (Figure 1).

Required staging investigations were in keeping with standard evidence-based practise in breast cancer management (including FBC, biochemical screen, CXR) and all patients had to have normal cardiac function (ECHO or MUGA) with further staging investigations only if clinically indicated. Inclusion criteria (Table 1) included age >70 years (either gender) and WHO performance status 0 or 1 with a diagnosis of invasive primary breast cancer, which had been surgically treated by wide local excision or mastectomy with clear margins (>1 m apart from deep margin if full thickness resection). Axillary staging was performed by sentinel node biopsy, axillary sampling or clearance with all patients found to be node positive having had axillary clearance or radiotherapy to the axilla. In addition, patients had to have been assessed as being at high risk of relapse within 5 years, indicated by usual prognostic factors of tumour size, grade, lymph node status and ER score. Other constraints were that patients had to be fit to receive chemotherapy, if allocated, including adequate bone marrow, hepatic and renal function and no active infection.

Patients were required to give written informed consent; randomisation was to take place as soon as reasonably possible after definitive surgery (ideally within 8 weeks) and the patient was to be available for routine long-term hospital follow-up.

Previous exposure to anthracycline chemotherapy or mantle radiotherapy at any time required exclusion as did inoperable breast cancer (T4 and/or N3 disease), a contraindication to or patient choice not to have radiotherapy following breast-conserving surgery. Patients were not eligible if they had had a previous invasive breast cancer, DCIS treated systemically or any other systemic therapy within the last 5 years. Patients with any previous haematological malignancy or melanoma at any time were also ineligible.

Inclusion of the first 200 patients was designated the pilot phase and aimed to test the viability of recruiting patients in the 70+ year age group to a trial of chemotherapy *vs* no chemotherapy and to evaluate the tolerability and acceptability of treatment. An accrual rate of ∼25 patients per month, a patient acceptance rate of 25% and 200 patients recruited within 1 year would have indicated viability of continuing to the full study. The pilot phase also included a comprehensive quality of life study.

**Figure 2:**
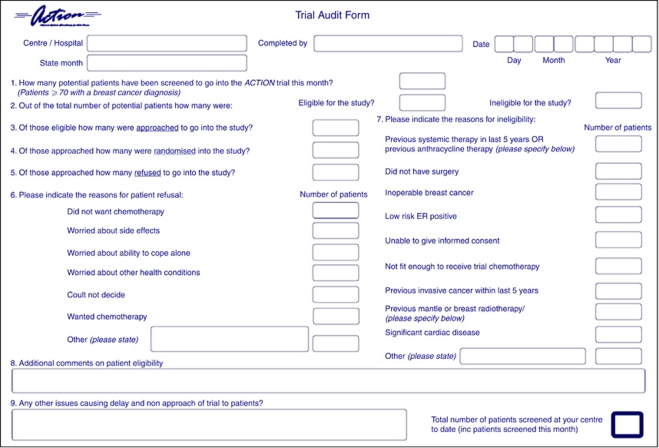
Screening log.

Throughout the pilot phase, centres were asked to voluntarily complete detailed screening logs of all patients aged over 70 who had received primary surgery for invasive breast cancer (Figure 2). Anonymised data on reasons for ineligibility and reasons for patients declining study entry were collected regularly, allowing a review of eligibility criteria. Results of the pilot phase were to be reviewed by an Independent data Monitoring Committee to inform the design and viability of the main trial.

Following successful completion of the pilot phase, the primary endpoint was to be the relapse-free survival interval (RFI), including as events any local or distant relapse, contralateral and ipsilateral breast second primary cancers, breast cancer deaths at any time and all deaths within 4 months or randomisation. The rationale for the choice of endpoint was to evaluate the most sensitive assessment of breast cancer outcome whilst ensuring that any early excess in mortality during treatment was duly incorporated. Secondary endpoints included disease-free survival (for completeness and comparison with other studies), overall survival, compliance, safety and tolerability of chemotherapy and patient-assessed quality of life.

In addition, a biological study was proposed to investigate markers of resistance to chemotherapy, to provide a basis for exclusion of patients from ineffective treatment in the future. This trial population was likely to provide one of the few remaining opportunities to study the natural history and biology of breast cancer in an elderly population as trials in breast cancer comprising a no-treatment arm, irrespective of the age of the patient, are becoming less acceptable to patients. In the best interests of patients, more trials are focusing on gathering biological evidence for exclusion of patients from treatment with agents that may result in toxicity but not efficacy.

The statistical assumptions underlying the ACTION trial design were that the relapse rate in the control arm within 5 years would be 30% in this ER negative/weakly positive disease group. A total of 1000 patients were to be recruited over a 3-year period in the main trial in order to have 80% power to detect an increase in RFI from 70% to 77.9% (α 0.05, two sided).

## Results

### Early feasibility exercises

Responses to a questionnaire circulated to UK breast oncologists on 29 April 2003 indicated a high level of interest in a trial testing the efficacy of adjuvant chemotherapy for older patients. Completed questionnaires were received from 89 clinicians at 56 UK centres, the majority supporting a trial design comparing anthracycline polychemotherapy with no chemotherapy. This widespread and enthusiastic support encouraged the development of the protocol and supported the successful application for research funding (CTAAC, Cancer Research UK – CRUK/06/002).

#### Feasibility of ACTION

The ACTION trial opened in July 2007, with 43 centres opening to recruitment over the subsequent 18 months.

In the first year of recruitment 92 screening logs were returned from 23 centres. A total of 272 patients had been screened of whom 21 (7.7%) were eligible for the trial. In all, 19 out of 21 eligible patients were approached for trial entry and two had been randomised (Table 2). Due to the voluntary nature of the screening logs it is likely that the figure underestimates the number of patients directly approached to enter the ACTION trial. Of those patients approached who declined trial entry, most lacked uncertainty over whether or not to undergo chemotherapy, 1 patient chose to receive chemotherapy and 10 elected not to receive chemotherapy (Table 3). The most common reason for ineligibility related to ER score (Table 4).

Although the inclusion criteria relating to ER status (i.e., ER negative or weakly positive) had been devised to restrict the trial to those at high risk of early relapse, screening logs and anecdotal feedback suggested the restriction rendered ineligible a number of patients who would be deemed at high risk of relapse irrespective of their ER status (i.e., extensive nodal involvement, grade 3 etc). In June 2008, therefore, a protocol amendment was implemented to widen the eligibility criteria to more pragmatically include patients assessed by their clinician to be at high risk of early breast cancer relapse irrespective of hormone receptor status. Additionally screening data led to agreement to allow inclusion of those with previous solid tumours more than 5 years ago and HER2-positive patients, with an allowance for herceptin to be given as per local practise if deemed appropriate.

In the time between the protocol amendment and centres receiving local approval of the change in eligibility, 27 screening logs were returned from 17 centres with 79 patients screened. An additional two patients were randomised to the study during this period. After the eligibility changes were implemented a further 54 screening logs were received from 27 centres with a total of 151 patients screened. A higher proportion of patients were eligible for the trial as expected following the amendment (21 out of 151 (13.9%)) with 18 patients approached and no randomisations. Again, the majority declined because they elected not to have chemotherapy (13 patients). Patients who had ER-positive breast cancer but who were deemed at low risk of early relapse were the majority of those ineligible (Tables 1, 2, 3).

In November 2008, a decision was made by the Trial Management Group, endorsed by the independent Trial Steering Committee and the trial's Sponsors, to close to further recruitment after the organisation providing research funding chose not to award the next annual installment of the grant on the grounds of lack of viability. Follow-up was also terminated in the four randomised patients.

## Discussion

An audit carried out at Southend General Hospital between 1 April 2004 and 30 September 2004 aimed to quantify the likely size of the eligible trial population, and to evaluate the acceptability and tolerability of four 3-weekly cycles of doxorubicin 60 mg m^−2^ and cyclophosphamide 600 mg m^−2^ in breast cancer patients over 70 years of age. A total of 54 patients with early breast cancer (median age=78.0 years (IQR=74–83)) were identified. In all, 52% lived alone and 61% had co-morbid conditions (Johnson *et al*, 2005).

In all, 17 (31%) did not receive primary breast surgery, the majority of whom (82%) were ER positive. Those who did not undergo surgery were significantly older than those who did undergo surgery (mean age 83.2 years (s.d. 6.6); *P*⩽0.001), and there was a suggestion of an association between not having surgery and living alone (no surgery+living alone=12 (71%) *vs* surgery+living alone=16 (43%); *P*=0.06). Using the Nottingham Prognostic Index (NPI) score, 17 (46%) of the 37 patients undergoing surgery scored 4.4 or more, giving them a high risk of relapse within 5 years. They would, therefore have been eligible for ACTION. During the period of the audit, chemotherapy was offered postoperatively to patients with at least one high risk factor. A total of 13 patients were offered chemotherapy; 11 of whom had an NPI>4.4. Of those with NPI>4.4 who were not offered chemotherapy, two were considered too frail, and the reason for the MDT decision for the remaining four was not specified. In all, 6 of the 13 patients (46%) offered chemotherapy accepted it. Those accepting chemotherapy had a mean age of 73.1 (s.d. 2.3) years. One (17%) lived alone and two (33%) had co-morbidity. Treatment was well tolerated (no grade 3/4 toxicity), and no patients required dose reductions or delays. Prophylactic GCSF was not given and no patient required hospitalisation. Although the numbers were small, the Southend audit suggested that an anthracycline-based chemotherapy regimen could be delivered safely to selected patients aged 70+ with a high risk of breast cancer relapse. Age and failure to undergo surgery are the factors most likely to limit recruitment.

Given the level of enthusiasm identified before the launch of ACTION, its failure to recruit sufficient patients to establish its viability was particularly disappointing. There are number of reasons, which contributed to this outcome. First, and in contrast to the pre trial audit, a large number of patients who were screened proved to be ineligible. The great majority of these were due to the patients not being at high risk of recurrence within 5 years of diagnosis due to a high ER score or low grade of tumour. Other significant reasons included previous invasive cancer within the past 5 years or patients deemed not fit for chemotherapy. Only a small number of patients did not undergo surgery although there may have been other patients who were not offered surgery in their referring centres as the screening process was not comprehensive. More than 400 patients were screened and 80% were ineligible for the reasons given above. This proportion was much greater than predicted by the preoperative audit and effectively demonstrated that a clinical trial aiming to recruit more than 1000 patients over a 3-year period was not feasible. A patient information sheet was provided to patients following their diagnosis and surgery. This acknowledged that there was a perception of chemotherapy being associated with increased toxicity in older patients. However, it also stated that with modern chemotherapy this perception may not be correct. We also said that we were selecting women over the age of 70 who were in generally good health for this trial. This we felt would reassure patients.

Unfortunately, the majority of the patients who were eligible and were approached declined trial entry. In most cases this was due to the patient not wishing to undergo chemotherapy although a smaller proportion made an active choice for chemotherapy. This study was not designed to investigate the patient's reasons for declining or accepting chemotherapy, but this is the subject of a current study (AChEW: Ballinger *et al*, personal communication). A subsequent non-randomised study, which seeks to correlate the results of standard geriatric assessment tests with the toxicity of subsequent chemotherapy is also planned (ACTION2) This will enlarge upon the information gathered on patient's attitudes to chemotherapy in the elderly breast cancer patient.

Randomised trials that involve a comparison of significant toxic complications with none have, not surprisingly, always presented difficulties in recruitment. When provided with more complete information about the benefits and risks of the different treatment options before agreeing to randomisation the patients made their own active, informed decisions. This is a well-recognised problem in non-blinded randomised trials where the control group does not receive active treatment. This is not likely to be due to a specific feature of breast services in the United Kingdom as the no treatment control arm of the European CASA trial also failed, recruiting only eight patients. In the CASA trial the options comparing standard chemotherapy with low-dose metronomic chemotherapy faired slightly better recruiting ∼60 patients but overall recruitment was inadequate and the trial also closed. More recently a North American study comparing standard chemotherapy (EC or CMF) with single agent capecitabine but without a no treatment control group, successfully recruited more than 600 patients and demonstrated significant disease-free survival benefit in the standard chemotherapy arm (Muss *et al*, 2009). Furthermore the two mortalities in the study were in the experimental group and overall toxicity in the standard chemotherapy arm was acceptable. Drawbacks of this study included the recruitment of a significant proportion of patients between the age of 65 and 70, and the fact that capecitabine proved to be a rather toxic control arm. However it does demonstrate that older patients are willing and able to undergo chemotherapy within the context of a randomised trial. Further evidence for the problems associated with non-blinded randomised controlled trials comes from the early closure of the ESTEeM trial evaluating the role of surgery in frail elderly patients with primary operable breast cancer. In this study, which compared primary endocrine therapy with surgery and adjuvant endocrine therapy in patients over the age of 75, there was no shortage of eligible patients. However despite initially indicating a willingness to join the trial the great majority of patients opted for one or other treatment (∼60% surgery and 40% primary endocrine therapy) and subsequently declined randomisation. This adds further evidence to the increasing desire for older patients to receive comprehensive information and to plan an active role in decisions relating to treatment.

The chemotherapy trials listed above lack a no-treatment control arm, so that the survival effect of the intervention is not measurable on the natural history of the disease. They also do not address the issue of patient fitness for treatment, which will be the purpose of the next ACTION trial (ACTION2).

## Conclusion

The pilot phase of the ACTION trial almost certainly represents the last attempt in breast cancer to compare chemotherapy against no chemotherapy within the context of a randomised controlled trial. A similarly designed trial run via the IBCSG (CASA study), also folded due to low recruitment, suggesting that the experiences of the United Kingdom are not unique. There is a need to explore other approaches to evaluate the risks and benefits of chemotherapy in selected older patients. Alternative strategies, including investigating the use of both qualitative geriatric assessment tests and quantitative biological tests in a non-randomised trial for the elderly breast cancer patients are under development. The question of selecting older patients for adjuvant chemotherapy will become even more important in our ageing Western populations.

## Figures and Tables

**Table 1:** Principal inclusion/exclusion criteria

**Inclusion criteria**	**Exclusion criteria**
• Aged over 70 • Male or female • WHO performance status 0 or 1 • Histological diagnosis of early stage invasive breast carcinoma • Primary operable breast cancer surgically treated by wide local excision or mastectomy with clear margins • Axillary staging performed (node-positive patients to have axillary clearance or radiotherapy to the axilla) • Fit to receive chemotherapy • Adequate bone marrow, hepatic and renal function • No active, uncontrolled infection • Written informed consent • Available for routine long-term hospital follow-up	• Previous invasive breast cancer within the last 5 years • Previous DCIS within the last 5 years if treated systemically • Previous haematological malignancy or melanoma • Chemotherapy within the last 5 years • Previous anthracycline chemotherapy at any time • Primary inoperable breast cancer (T4 and/or N3 disease) • Breast-conserving surgery with no plans for postoperative radiotherapy • Previous mantle radiotherapy • Significant cardiac disease • Unable or willing to give informed consent
	
*Changes made in July 2008*	
High risk of relapse within 5 years ER negative or ER weakly positive (Allred score ⩽5)	Any previous systemic anti-cancer therapy, or any solid tumour in the last 5 years
	
*was changed to*	*was changed to*
High risk (∼30%), includes patients with HER2-positive disease and/or ER-negative disease, or ER positive with grade 3 disease and/or 4+ positive nodes	No previous anthracycline chemotherapy at any time, and no systemic anti-cancer therapy within the last 5 years

Abbreviations: DCIS, ductal carcinoma *in situ*; ER=oestrogen receptor; WHO=World Health Organization.

Randomisation was as soon as reasonably possible after definitive surgery, ideally within 8 weeks.

**Table 2:** Summary of the screening data received before, during and after the change in eligibility

	**Screening data received before June 2008**		**Screening data received between June 2008 and local R&D approval**		**Screening data received after local R&D approval**		**Total**	
	***n***	**%**	***n***	**%**	***n***	**%**	***n***	**%**
Total patients screened	272	100	79	100	151	100	502	100
Total eligible	21	7.7	6	7.6	21	13.9	48	9.6
Total approached	19	7	6	7.6	18	11.9	43	8.6
Total randomised	2	0.7	2	2.5	0	0	4	0.8
Total refused	17	6.3	4	5.1	18	11.9	39	7.8
Total ineligible	248	91.2	73	92.4	125	82.8	446	88.8
Total unknown eligibility	3	1.1	0	0	5	3.3	8	1.6

Abbreviation: R&D=research and development committee.

**Table 3:** Reasons for eligible patients declining to take part in ACTION

	**Screening data received before June 2008**		**Screening data received between June 2008 and local R&D approval**		**Screening data received after local R&D approval**		**Total**	
	***n***	**%**	***n***	**%**	***n***	**%**	***n***	**%**
Number of eligible patients who refused trial	17	100	4	100	18	100	39	100
Did not want chemotherapy	10	58.8	4	100	13	72.2	27	69.2
Wanted chemotherapy	1	5.9	0	0	4	22.2	5	12.8
Worried about side effects	2	11.8	0	0	1	5.6	3	7.7
Wanted to make own decision (no randomisation)	1	5.9	0	0	0	0	1	2.6
Unknown	3	17.6	0	0	0	0	3	7.7

Abbreviation: R&D=research and development committee.

**Table 4:** Reasons for ineligibility for the ACTION trial

	**Screening data received before June 2008**		**Screening data received between June 2008 and local R&D approval**		**Screening data received after local R&D approval**		**Total**	
	***n***	**%**	***n***	**%**	***n***	**%**	***n***	**%**
Number of ineligible patients	248	100	73	100	125	100	446	100
Low risk ER+/Allred or quick score >5	126	50.8	33	45.2	43	34.4	202	45.3
Previous invasive cancer/metastatic disease	39 (metastatic=6)	15.7	9 (metastatic=1)	12.3	14 (metastatic=2)	11.2	62 (metastatic=9)	13.9
Not fit enough to receive trial chemotherapy	23	9.3	12	16.4	20	16	55	12.3
HER2 positive	18	7.3	0	0	0	0	18	4
Did not have surgery	4	1.6	2	2.7	9	7.2	15	3.4
Inoperable breast cancer	4	1.6	3	4.1	0	0	7	1.6
Significant cardiac disease	4	1.6	2	2.7	1	0.8	7	1.6
Previous systemic therapy	0	0	0	0	5	4	5	1.1
Unable to give informed consent	2	0.8	1	1.4	0	0	3	0.7
More than 8 weeks since surgery	2	0.8	0	0	0	0	2	0.4
Other	26	10.5	11	15.1	33	26.4	70	15.7
Investigator decision – not to give chemotherapy	6	2.4	6	8.2	7	5.6	19	4.3
DCIS	6	2.4	0	0	4	3.2	10	2.2
Benign disease	4	1.6	0	0	2	1.6	6	1.3
Further surgery required	2	0.8	1	1.4	2	1.6	5	1.1
Investigator decision – to give chemotherapy	4	1.6	0	0	0	0	4	0.9
Patient did not want active treatment	0	0	1	1.4	1	0.8	2	0.4
Neoadjuvant chemotherapy given	0	0	0	0	2	1.6	2	0.4
No axillary surgery	1	0.4	0	0	0	0	1	0.2
Investigator decision – not otherwise specified	0	0	0	0	1	0.8	1	0.2
Paget's disease of nipple only	0	0	1	1.4	0	0	1	0.2
Bilateral breast cancer	0	0	0	0	1	0.8	1	0.2
Pathology implies lymphoma	0	0	0	0	1	0.8	1	0.2
Node-negative disease	0	0	0	0	1	0.8	1	0.2
Patient did not attend clinic	0	0	1	1.4	0	0	1	0.2
Patient did not want to go in any clinical trial	0	0	1	1.4	0	0	1	0.2
Patient in another trial	0	0	0	0	1	0.8	1	0.2
Awaiting test results	1	0.4	0	0	0	0	1	0.2
Unknown	2	0.8	0	0	10	8	12	2.7

Abbreviations: ER=oestrogen receptor; R&D=research and development committee.
